# Effects of Ionizing Irradiation on Mouse Diaphragmatic Skeletal Muscle

**DOI:** 10.3389/fphys.2017.00506

**Published:** 2017-07-25

**Authors:** Tingyang Zhou, Lanchun Lu, Shiyong Wu, Li Zuo

**Affiliations:** ^1^Radiologic Sciences and Respiratory Therapy Division, School of Health and Rehabilitation Sciences, The Ohio State University College of Medicine Columbus, OH, United States; ^2^Interdisciplinary Biophysics Graduate Program, The Ohio State University Columbus, OH, United States; ^3^Department of Radiation Oncology, The Ohio State University James Cancer Hospital Columbus, OH, United States; ^4^Edison Biotechnology Institute, Ohio University Athens, OH, United States; ^5^Molecular and Cellular Biology Program, Department of Chemistry and Biochemistry, Ohio University Athens, OH, United States

**Keywords:** radiotherapy, C2C12 cells, reactive oxygen species, oxidative stress, skeletal muscle

## Abstract

Undesirable exposure of diaphragm to radiation during thoracic radiation therapy has not been fully considered over the past decades. Our study aims to examine the potential biological effects on diaphragm induced by radiation. One-time ionizing irradiation of 10 Gy was applied either to the diaphragmatic region of mice or to the cultured C2C12 myocytes. Each sample was then assayed for muscle function, oxidative stress, or cell viability on days 1, 3, 5, and 7 after irradiation. Our mouse model shows that radiation significantly reduced muscle function on the 5th and 7th days and increased reactive oxygen species (ROS) formation in the diaphragm tissue from days 3 to 7. Similarly, the myocytes exhibited markedly decreased viability and elevated oxidative stress from days 5 to 7 after radiation. These data together suggested that a single dose of 10-Gy radiation is sufficient to cause acute adverse effects on diaphragmatic muscle function, redox balance, and myocyte survival. Furthermore, using the collected data, we developed a physical model to formularize the correlation between diaphragmatic ROS release and time after irradiation, which can be used to predict the biological effects of radiation with a specific dosage. Our findings highlight the importance of developing protective strategies to attenuate oxidative stress and prevent diaphragm injury during radiotherapy.

## Introduction

Radiation therapy (RT) is one of the three major treatment options (surgery, chemotherapy, RT) for cancers. The primary side effect associated with RT can be unintentional radiation damage to normal tissues and organs surrounding the tumor target (Stewart and Fajardo, 1971; Van Der Kogel and Barendse, 1974; Hishikawa et al., 1984; Delanian et al., 1994; Dunlap et al., 2010). Lung cancer, breast cancer, and stomach cancer are among the most commonly diagnosed and lethal cancers worldwide (Ferlay et al., 2010; Torre et al., 2015). For those patients, radiation treatment can potentially damage the chest wall and diaphragm. Although there has been research into sparing the amount of normal tissue that is exposed to radiation during RT, including Stereotactic Body Radiotherapy and held-breath self-gating techniques, complications are still evident in the precision of RT due to poor image resolution, improper scanner calibration, and motion artifacts (Kim et al., 2001; Jiang, 2006; Schweinitz, 2012; Li et al., 2016). The diaphragm is the most important respiratory muscle that may be frequently exposed to radiation during RT due to its vicinity to tumor-susceptible organs (e.g., breast and lungs) and constant movement during respiration (Vedam et al., 2003; Jiang, 2006; McCool and Tzelepis, 2012; Ausili Cefaro, 2013). Furthermore, conventional RT typically includes anisotropic margins surrounding the tumor volume to ensure the coverage of clinical target volume (Vedam et al., 2003). In cases where the tumor extends into the diaphragm, direct irradiation of the diaphragm is necessary (Schweinitz, 2012). These factors may increase the risk of diaphragm injury from RT. However, potential physiological alterations in the diaphragm following radiation exposure have not been thoroughly evaluated.

In most occurrences, a decline in diaphragm efficiency is not life-threatening due to its tough nature as skeletal muscle (Laroche et al., 1988; Prezant et al., 1990). However, compromised diaphragm function is linked to dyspnea, reduced exercise tolerance, and sleep-disordered breathing (Hart et al., 2002; McCool and Tzelepis, 2012). This adds burden to normal physical activities and negatively affects quality of life (McCool and Tzelepis, 2012). In particular, diaphragm dysfunction may be associated with various respiratory diseases, such as chronic obstructive pulmonary disease (COPD). In fact, diaphragm weakness-induced hypercapnic respiratory failure may be one of the leading causes of death in the final stages of COPD (Ottenheijm et al., 2005). Ionizing radiation (IR) is known to promote reactive oxygen species (ROS) production, which potentially contributes to tissue damage (Riley, 1994). ROS are naturally generated within biological systems, and play critical roles in mediating immune response, muscle function, conditioning, and cellular stress responses (Zuo et al., 2013, 2015a). Nevertheless, under pathophysiological conditions or cellular stresses, ROS formation is appreciably elevated, which, if levels exceed antioxidant defense capacities, can lead to oxidative stress and a decline in muscle function (Riley, 1994; Zuo et al., 2015b). Over-accumulation of ROS have been implicated in a variety of diseases including neurodegenerative disorders, respiratory diseases (e.g., COPD and asthma), sepsis-induced diaphragm dysfunction, and myocardial ischemic reperfusion injuries (Cairoli et al., 1982; Phelan and Gonyea, 1997; Lin et al., 1998; Barton-Davis et al., 1999). For instance, when heart experiences ischemia, myocardium rapidly generates ROS which is further exacerbated during reperfusion thus resulting in substantial myocardial injuries (Cairoli et al., 1982). Additionally, sepsis-induced diaphragm dysfunction has been suggested to be mediated by reactive species as the inhibition of inducible isoform of NO synthase considerably attenuates myofiber injuries and muscle dysfunction (Lin et al., 1998). In response to sepsis, levels of inflammatory cytokines are drastically elevated, initiating excessive ROS formation in organs such as the diaphragm (Callahan and Supinski, 2009). Over-accumulation of ROS lead to mitochondrial impairment in skeletal muscles (Callahan and Supinski, 2009; Peruchi et al., 2011). Further evidence has shown that ROS are potentially involved in the activation of caspase and calpin pathways, contributing to muscle cell apoptosis and protein loss (Callahan and Supinski, 2009). These findings highlight the critical roles of ROS in regulating respiratory muscle function, indicating that ROS are tightly associated with redox balance and survival of myocytes (Smith et al., 2000; Circu and Aw, 2010). In this study, we aim to evaluate the potential side effects induced by IR on the diaphragm. Following a 10 Gy-IR treatment plan, we evaluated diaphragmatic function, myocyte viability and ROS production in both animal and cell models. We also established associated physical models in order to understand and predict both property and functional alteration in the diaphragm. This physical model can potentially help to extrapolate the potential side effects of specific IR dosages on the diaphragm, which will facilitate the development of a more effective and secure RT plan in clinical settings.

## Materials and methods

### Mice irradiation

Prior to irradiation treatments, 24 mice (C57BL6, male, 12 weeks old) were anesthetized via intraperitoneal injection of ketamine (70 mg/kg) and xylazine (10 mg/kg). Following complete anesthesia, mice were placed under the x-ray beam collimator in a supine position; the diaphragmatic area of each mouse was exposed to a one-time vertical irradiation at a 600 cGy/min dose rate, and 6 MV energy of 10 Gy using the Varian TrueBEAM Linear Accelerator (Varian Medical Systems, Palo Alto, California, USA). The irradiation field size on a mouse was set to 20 × 3.5 mm to cover the entire diaphragm and minimize the exposure of radiation to normal tissues surrounding the diaphragm. Control mice followed the same protocol, but did not receive radiation. This study was carried out in accordance with the recommendations of the Institutional Animal Care and Use Committee (IACUC) of the Ohio State University. The protocol was approved by IACUC (#: 2013A00000046-R1).

### Diaphragm function and ROS analysis

**Figure 1:**
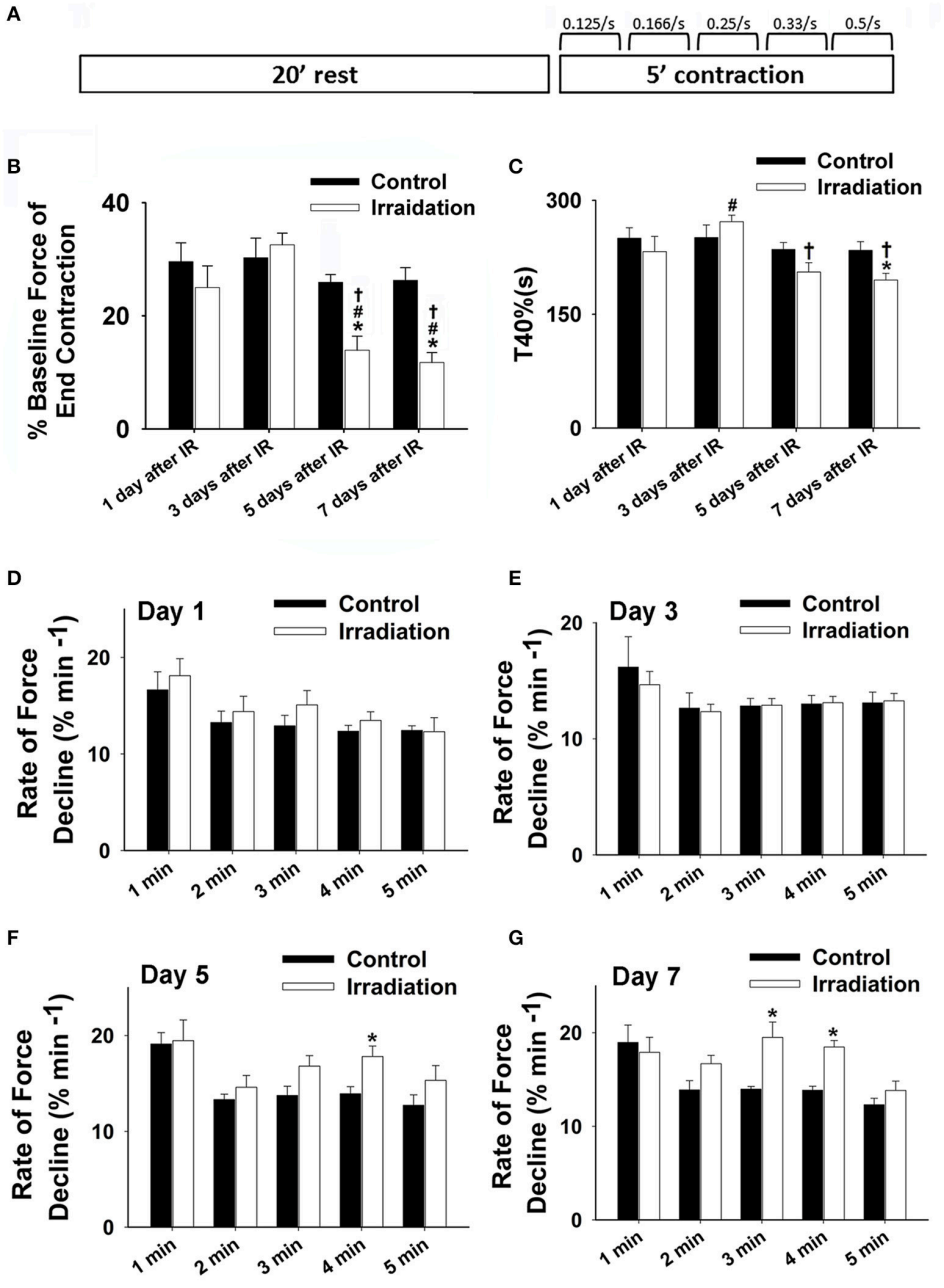
**(A)** Schematic showing muscle function protocol. **(B)** Grouped data showing the percentage of baseline force at the end contraction (300 s) in irradiation vs. control group on the 1st, 3rd, 5th, and 7th days after IR (*n* ≥ 6 for all treatment groups). **(C)** Grouped data of T40% (s) in irradiation vs. control group on the 1st, 3rd, 5th, and 7th days after IR (*n* ≥ 6 for all treatment groups). **(D–F)** Grouped data showing the rates of force reduction during the 5-min contractile period from the initial force (% min^−1^) in irradiation vs. control group on day 1 **(D)**, 3 **(E)**, 5 **(F)**, and 7 **(G)** after IR (*n* ≥ 6 for all treatment groups). ^*^Significant difference from control at the same day/time point; ^#^Significant difference from 1 day after IR within the same treatment; ^†^Significant difference from 3 days after IR within the same treatment. Sig. (2-tail); *p* < 0.05.

On each of the 1st, 3rd, 5th, and 7th days after IR, three irradiated mice and 3 control mice were sacrificed for *in vitro* muscle function analysis. After complete anesthesia using ketamine and xylazine, the diaphragms of the mice were immediately isolated and preserved in Ringer's solution bubbled with 95% O_2_ and 5% CO_2_. Two or three muscle strips were dissected out from each diaphragm. Isolated muscle strips were then mounted in a contraction chamber (model 800MS; Danish Myo Techonology, Aarhus, Denmark). The central tendon of the muscle strip was secured to a mobile lever, which was designed to adjust the muscle to its optimal length where the maximal force was generated. This initial maximal contraction force was used as the baseline for force normalization. This calibration of muscle force is necessary to exclude the potential interference caused by individual surgical technique or mouse variation. The opposite end was fixed on a stationary force transducer (Roberts and Zuo, 2012). After the optimal length was attained, the muscles were allowed to equilibrate at room temperature for 20 min, followed by a 5-min tetanic contraction at 37°C. Muscles were electrically stimulated (S48 stimulator; Grass Technologies, West Warwick, RI) for 5 min with increasing train frequencies of 0.125, 0.166, 0.25, 0.33, and 0.5 contractions per sec for each min using square-wave pulses (0.5-ms pulse duration, 250-ms train duration, 30 V, 70 Hz) (Figure 1A). The A-D board (model ML826; AD instruments) was employed to convert the analog signals to digital data which was further analyzed using LabChart 7.3.1 software (AD Instruments, Sydney, Australia) (Zuo et al., 2011, 2014; Roberts and Zuo, 2012). The contractile force at the initial (1 s) and end (300 s) of the contraction period was collected and normalized by the baseline contractile force.

To understand the redox mechanisms mediating IR-induced diaphragm injury, we also determined the rate of muscular ROS formation during the contractile period on days 1, 3, 5, and 7 after IR. Cytochrome *c* (cyt *c*, 5 μM, Sigma-Aldrich, MO, USA), an extracellular ROS probe, was employed to detect ROS levels in muscle superfusate. Superoxide dismutase (SOD, an extracellular superoxide (${\text{O}}_{2}^{•-}$) scavenger, 200 U/mL, Sigma-Aldrich, MO, USA) was added to the superfusate to scavenge ROS as negative control. Cyt *c*, when reduced by ${\text{O}}_{2}^{•-}$, displays an increased absorbance at 550 nm, which can be measured by a spectrophotometer (Nanodrop 2000, Thermal Scientific, MA, USA). The absorbance at 540 nm and 560 nm was averaged and calculated as the baseline subtracted from the peak absorbance at 550 nm (Zuo et al., 2000, 2014). An extinction coefficient of 18.5 × 10^3^ M^−1^cm^−1^ was used to determine ROS concentration. Cyt *c* reduction rate (ROS generation rate, nmol•min^−1•^mg^−1^ dry wt) was calculated by dividing the change in cyt *c* reduction during the 5-min contractile period by muscle dry wt and by 5 min (Kolbeck et al., 1997; Zuo et al., 2014).

### Cell irradiation, ROS and viability mesurement

Fused myotubes derived from mouse C2C12 myoblast cell line (Sigma-Aldrich, MO, USA) were cultured on 24-well plate in Dulbecco's Modified Eagle Medium or DMEM (Life Technologies, CA, USA) with 10% fetal bovine serum (FBS, Life Technologies, CA, USA) and 1% penicillin (Life Technologies, CA, USA) two days before irradiation treatment. The cells in the IR group received one-time irradiation at a 600 cGy/min dose rate, and 6 MV energy of 10 Gy. The same radiation setup was employed as that used for irradiating diaphragms (described in the *Mice Irradiation* section). Control cells followed the same procedure without irradiation treatment. Extracellular ROS (${\text{O}}_{2}^{•-}$) release in each well was determined on days 1, 3, 5, and 7 following IR using cyt *c*. On the days of analysis, six wells of cells from each group (IR and control) were loaded with 5μM of cyt *c* for 5 min. In order to confirm that the measured cyt *c* signals were due to (${\text{O}}_{2}^{•-}$), SOD (200 U/mL) was added to half of the wells to scavenge excessive (${\text{O}}_{2}^{•-}$) and to act as a negative control. The reduction levels of cyt *c* in the cell culture medium were determined by spectrophotometer analysis (Nanodrop 2000, Thermal Scientific, MA, USA) before and after the 5-min loading period using the same approach as previously described in the *Diaphragm Function and ROS Analysis* section. The extracellular ROS formation rate was indicated by the change of cyt *c* reduction per min. Following ROS assay, cells were detached using trypsin and stained by Trypan blue (Life Techonolgies, CA, USA). Cell viability of each well was then assessed using Cellometer Mini (Nexcelom Bioscience, Lawrence, MA, USA), expressed as the proportion of live cells over total cells.

### Statistical analysis

All statistical data are expressed as means ± SE. Data were analyzed using one-way ANOVA with time or dosage as variables. Statistical differences between various treatment groups were interpreted and displayed via LSD *post-hoc* test using SPSS (IBM, NY, US). Student's *t*-test was used to compare treatment vs. control. *p* < 0.05 was regarded as statistically significant.

## Results

### Diaphragm function was reduced by IR

**Figure 2:**
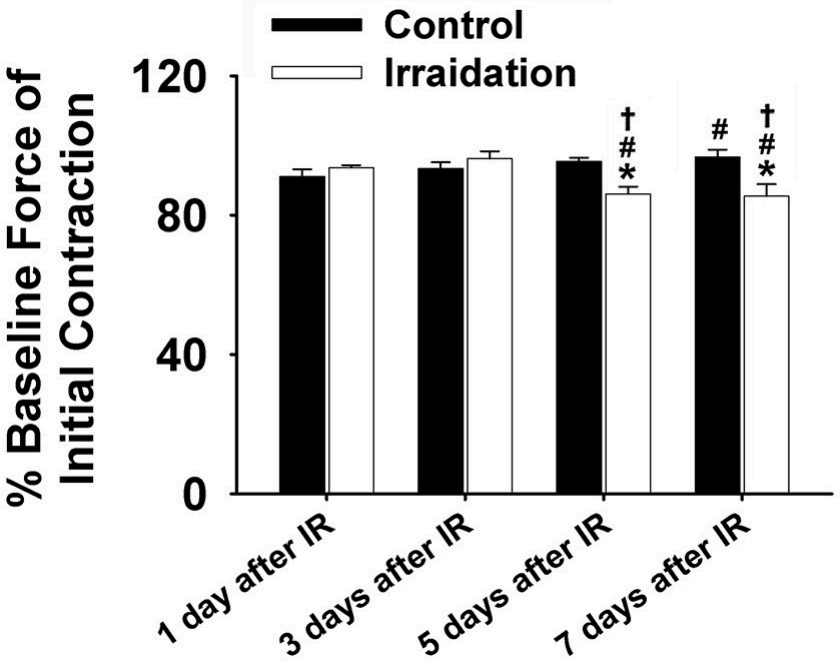
Grouped data showing the percentage of baseline force at the initial contraction (1 s) in irradiation vs. control group on the 1st, 3rd, 5th, and 7th days after IR (*n* ≥ 6 for all treatment groups). ^*^Significant difference from control at the same day/time point; ^#^Significant difference from 1 day after IR within the same treatment; ^†^Significant difference from 3 days after IR within the same treatment. Sig. (2-tail); *p* < 0.05.

Muscle function is expressed as the percentage of baseline force at the initial and the end of 5-min contraction as shown in Figures 2 and 1B, respectively. Dosage of 10 Gy-IR significantly compromised diaphragm function on day 5 and day 7 as compared to control. In addition, we defined the time to reach 40% of the first contraction force (T_40%_) as the “fatigue point”; the data are displayed in Figure 1C. T_40%_ decreased significantly in IR groups as compared to control on day 7. Interestingly, T_40%_ of the irradiated muscles on day 3 was greater than that on day 1 after IR (Figure 1C), which was likely due to the self-recovery mechanism of the diaphragm after irradiation. However, 5 and 7 days after IR, irradiation damage seemed to overcome this repair mechanism. Force decline rate for each train frequency was summarized in Figures 1D–G for days 1, 3, 5, and 7, respectively. There was no significant difference between IR and control groups in terms of fatigue tolerance on days 1 and 3 (Figures 1D,E). However, on the 5th and 7th days after IR, a higher rate of force decline was observed at ~3–4 min within the contractile period (Figures 1F,G). These data collectively indicate that 10-Gy IR treatment markedly weakened diaphragmatic muscle both 5 and 7 days after irradiation.

### IR induced ROS formation in diaphragm

**Figure 3:**
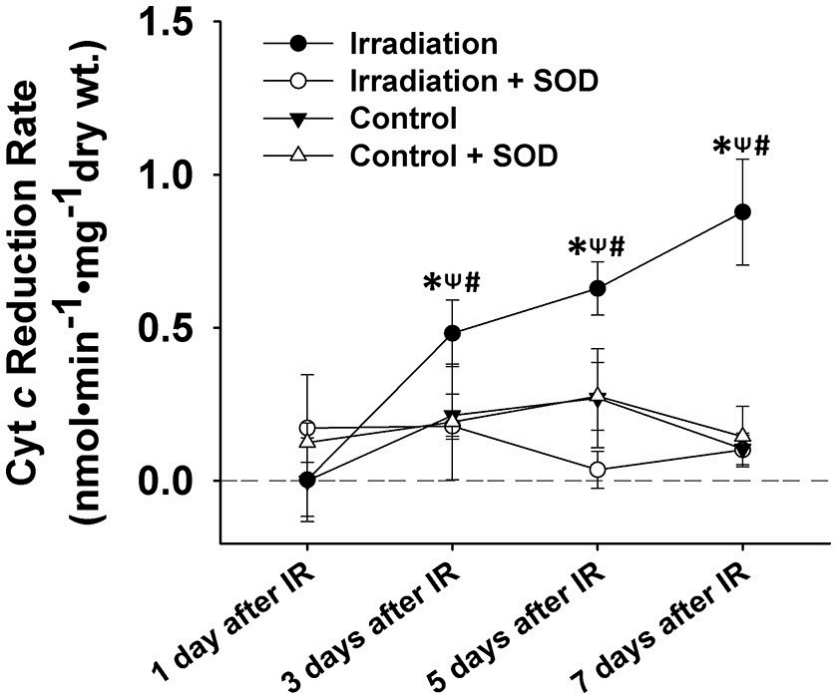
Grouped data showing the cyt *c* reduction rate (nmol•min^−1^•mg^−1^ dry wt) during the 5-min contractile period in the muscles of IR, IR + SOD, control, and control + SOD (*n* ≥ 3 for all treatments). All data were calibrated by subtracting the control value of day 1. ^*^Significant difference from control group at the same day; ^ψ^Significant difference from SOD group within the same treatment at the same day; ^#^Significant difference from 1 day after IR within the same treatment; Sig. (1-tail); *p* < 0.05.

ROS levels in control muscles were relatively constant on days 1, 3, 5, and 7. However, ROS production increased markedly from day 1 to day 7 in IR groups (Figure 3). Irradiated muscles released significantly higher levels of ROS during contractile periods than control muscles on days 3, 5, and 7 after IR treatment. The application of SOD in the superfusate effectively abolished this enhanced signal (Figure 3), confirming the specificity of cyt *c* for (${\text{O}}_{2}^{•-}$) in the study.

### IR induced ROS formation and lower viability in C2C12 cells

**Figure 4:**
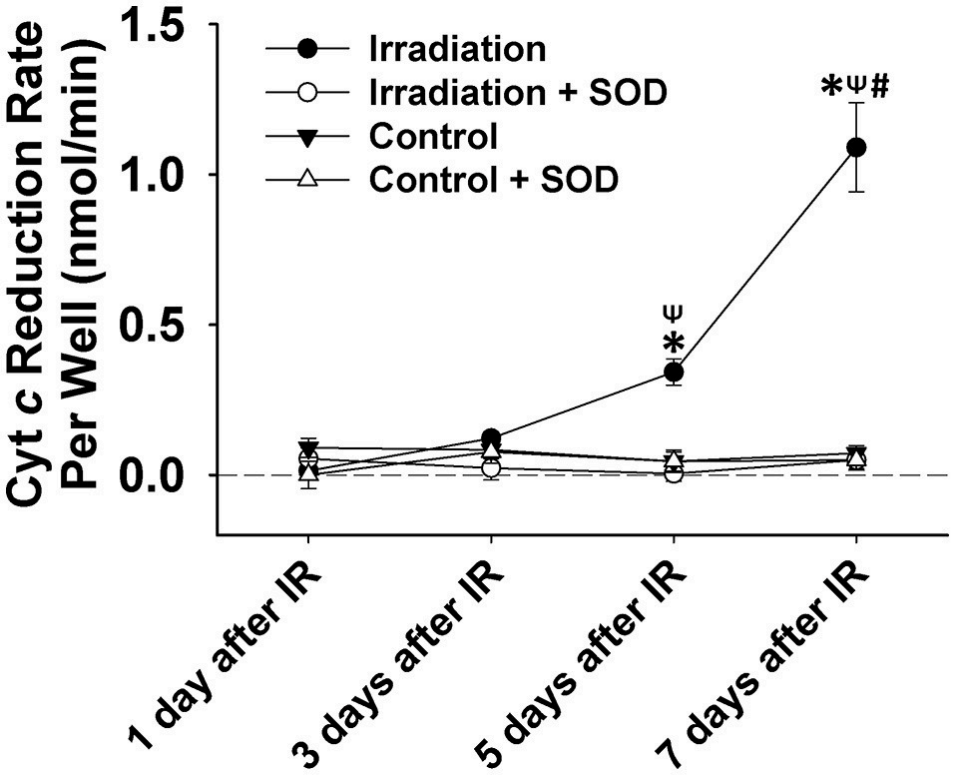
Grouped data showing the cyt *c* reduction rate per cell culture well (nmol•min^−1^) during a 5-min observation period in IR and control groups with and without SOD treatment on day 1, 3, 5, and 7 after IR (*n* = 6). All data were calibrated by zeroing the “control + SOD” value on day 1. ^*^Significant difference from both control and “irradiation + SOD” group on the same day; ^ψ^Significant difference from day 1 after IR within the same treatment; ^#^Significant difference from both day 3 and day 5 after IR of the same treatment. Sig. (2-tail); *p* < 0.05.

**Figure 5:**
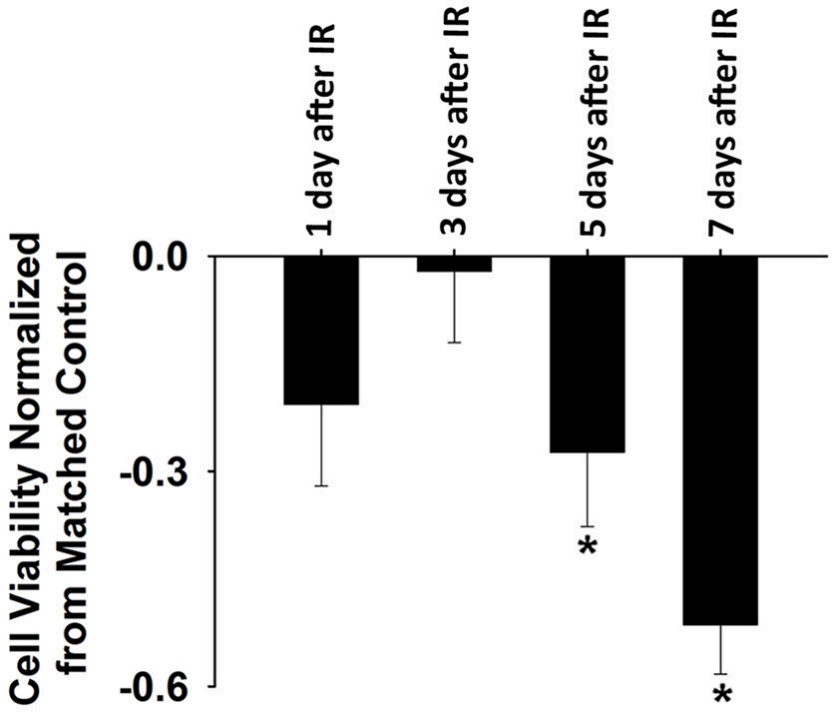
Normalized cell viability evaluated by Cellometer Mini on day 1, 3, 5, and 7 after IR. Each data was normalized by the corresponding control data based on the following equation: normalization = (IR-control)/control (*n* = 6 per treatment). ^*^Significant difference between IR treatment and matched control on the same day. Sig. (2-tail); *p* < 0.05.

IR treatment stimulated greater extracellular ROS release in C2C12 cells than in the control on the 5th and 7th days (Figure 4). SOD application in the culture medium effectively diminished this signal (Figure 4), again indicating the specificity of cyt *c* for ${\text{O}}_{2}^{•-}$. In addition, extracellular ROS formation demonstrated a marked increase from day 5 to day 7 after IR, while the control group showed little difference in extracellular ROS levels between days 1, 3, 5, and 7. Normalized cell viability data were grouped in Figure 5. The results showed that cell viability was significantly reduced on days 5 and 7 in IR groups in comparison with the control group (Figure 5).

### A physical model describing ROS production in diaphragm's response to IR

The production of ROS in cells and tissues during irradiation is involved with the complicated cell signaling and homeostasis processes. Thus, the exact mechanism of ROS production could not be sufficiently identified with available experimental data and measurements. However, we can empirically model and formularize the overall ROS production based on our current experimental data, which can be useful in describing the biological effects of ROS in response to certain irradiated doses. To achieve this, we proposed the following mathematical model to predictively quantify ROS levels produced over time under a prescribed irradiated dose. Simply using the existing data from a single dosage, it is convenient to extrapolate biological data of ROS formation for different doses, which can be clinically applied or confirmed in future studies. Based on the previous evidence showing that IR-induced ROS generation is time- and irradiated dose-dependent (Leach et al., 2001), we propose the following empirical formula that describes ROS production in relation to delivered doses and time after irradiation:

(1) $$\begin{array}{r} F\left(d,\;t\right)=A\left(d\right)\left(1-e^{-B\left(d\right)t}\right) \end{array}$$

where the two-dimensional function *F*(*d,t*) is dose- and time-dependent ROS formation; *d* is the dose, and t is the time after irradiation treatment. *A(d)* is a dose-dependent parameter that reflects the linear component of the irradiation-induced ROS formation, while dose-dependent parameter *B(d)* describes the overall effect of ROS production mediated by both exogenous sources (radiation) and endogenous sources (complex physiological responses such as metabolic and immune reactions of a mouse). *B(d)* is also assumed to be tissue-dependent. For a given dose, the two-dimensional function *F(d,t)* in formula (1) is simplified to a one-dimension function (time-dependent only) as shown in the following equation:

(2) $$\begin{array}{r} f\left(t\right)=A\left(1-e^{-Bt}\right) \end{array}$$

**Figure 6:**
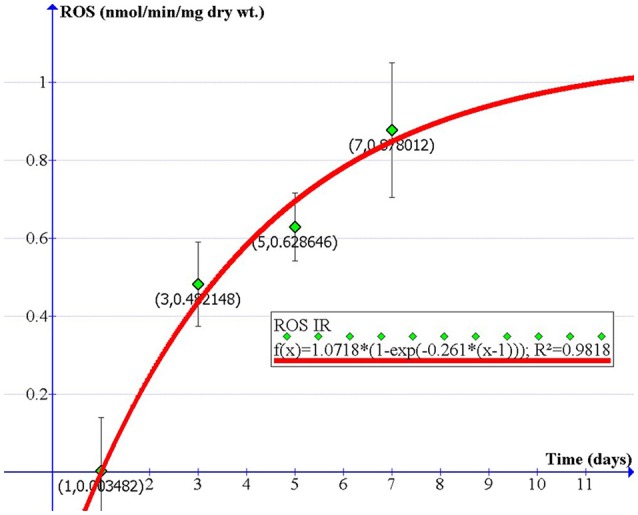
A physical model describing the rate of extracellular ROS production vs. time in diaphragm after exposure to 10-Gy IR (nmol/min/mg dry wt).

Our *in vivo* experiment provides us with ROS data generated from the diaphragm at four time points (1, 3, 5, and 7 days after 10-Gy irradiation). Parameters A (1.0718) and B (−0.261) were obtained by fitting our proposed model to the four data points. Thereby, the relationship between ROS formation in response to 10-Gy irradiation and the time after irradiation can be expressed in the equation below and in Figure 6.

(3) $$\begin{array}{r} f\left(t\right)=1.0718\times \left(1-e^{-0.261\left(t-1\right)}\right) \end{array}$$

where t is the time after irradiation. Using this model, we can reasonably predict the amount of ROS produced at any time after 10-Gy IR, which is potentially useful for RT planning and prognosis.

## Discussion

Our animal and cell radiation models suggest that 10-Gy IR exposure is sufficient to induce short-term muscle fatigue, cell death, and oxidative stress in the diaphragm. Specifically, we observed increased ROS formation in contractile diaphragm tissue on days 3–7 and in C2C12 cells on days 5–7 after radiation. While ROS can be beneficial to cellular processes, IR-induced oxidative stress damages DNA and muscle tissues, inhibits cellular division, and promotes tumor formation (Riley, 1994; Shackelford et al., 2000; Thomas and Darley-Usmar, 2000; Radak et al., 2008; Zuo et al., 2015b). Accordingly, we have developed a physical model to describe the relationship between diaphragmatic ROS release, radiation dose, and time. The established mathematical formula further allows us to quantifiably predict oxidative stress in the contractile diaphragm after IR exposure at various times after irradiation.

The results shown in Figure 3 (tissue) and Figure 4 (cell) clearly reflect that the change in ROS levels over time in the *in vivo* (mice) and *in vitro* systems (cells) is different. Although in both scenarios ROS increased over time after irradiation, the *in-vivo* and *in-vitro* systems showed different patterns of ROS production following the irradiation. This difference is likely due to the complex physiological processes that exist in an *in-vivo* system, such as immune response to the radiation. It may also be due to the differences in microenvironments between the *in vivo* and the *in vitro* systems. We noticed that for the first few days (i.e., days 1 and 3), *in-vivo* ROS production was greater than *in vitro*, possibly due to higher absolute cell numbers in the tissues than in the cell wells. After day 5, the *in-vivo* production of ROS decreased (relative to the *in vitro* model), which could likely indicate the activation of immune responses.

So far, scant research has been done to investigate the potential side effects of IR on the diaphragm. To our knowledge, this is the first study to thoroughly examine the effects of diaphragmatic irradiation of muscle function, cell viability, redox status, and physical modeling of ROS production using both mice and myocyte models. Due to its constant movement during respiration, the diaphragm can be frequently exposed to radiation during RT, which is delivered to the upper body to target tumors in breast, lung and liver cancers. In those diseases, radiation doses greater than 40 Gy are preferred to ensure local disease control and improvement of overall survival (Kong et al., 2006; Moran et al., 2014). Thus, we estimated a dosage of ~10 Gy to represent the off-target dose on the diaphragm and C2C12 cells in the current experiments. Based on our clinical experiences, this dose can exert marked effects on biological systems. In our cell model, 10 Gy-IR markedly decreased C2C12 cell viability and stimulated extracellular ROS release on days 5 and 7 (Figures 4, 5). Since elevated ROS levels play a key role in apoptotic activation, the reduced cell survival may be attributable to the IR-induced oxidative stress via apoptotic mechanisms (Powers et al., 2010). Similar results were reported by Hiseh et al., who investigated muscle function and oxidative stress in rat diaphragm in response to 5-Gy IR (Hsieh et al., 2016). They found that IR decreased diaphragmatic function and increased protein and DNA oxidation at 24 h after irradiation (Hsieh et al., 2016). These results are consistent with our findings that IR can lead to diaphragm dysfunction via the induction of oxidative stress (Hsieh et al., 2016). However, there are several differences between two studies. For example, decreased muscle force was observed on the 5th and 7th days in the current study, while it was observed on the 1st day by Hsieh et al. (2016). Indeed, we observed a trend of decline in muscle function on the 1st day after IR but it was not statistically significant (Figure 1). Likewise, IR-induced oxidative stress was evidenced in different time periods following irradiation. Hsieh et al. detected increased protein and DNA oxidation at 24 h, while our data suggested that IR caused more ROS release during days 3–7 (Hsieh et al., 2016). The discrepancy between these two studies can be potentially attributed to the differences in muscle contractile protocols, oxidative stress measurements, and radiation resistance between rats and mice. In particular, protein carbonyl and DNA oxidation is an indication of intracellular oxidative stress (Hsieh et al., 2016). However, cytochrome *c* used in the present study is an extracellular ROS probe. Since intra- and extracellular ROS have been suggested to be produced via different mechanisms (Hojan and Milecki, 2014), they were likely to be triggered by IR at different time points. Furthermore, cytochrome *c* is a probe specific for ${\text{O}}_{2}^{•-}$ (Hojan and Milecki, 2014). Other types of ROS such as H_2_O_2_ and ^·^OH may also be responsible for protein or DNA oxidation observed by Hsieh et al. at 24 h. Overall, it is evident that IR can induce oxidative stress and compromise diaphragm function. However, more work is needed to determine dosage levels and timeline for those IR-induced side effects on diaphragm muscles.

Muscle contraction is known to boost ROS production, which can promote fatigue development (Reid et al., 1992). In the control group, we observed a slight increase in ROS generation after 5-min muscle contraction; while the radiation-exposed muscles showed more significant ROS release on the 3rd, 5th, and 7th days than the control (Figure 3). The enhanced ROS formation indicates an imbalanced redox status after IR exposure, which may partially account for the declining muscle function observed on days 5 and 7 (Figure 1). Under physiological conditions, the primary sites of ROS formation in skeletal muscle include the mitochondria, transverse tubules, and sarcoplasmic reticulum. IR is capable of ionizing intra- or extracellular water to form ROS or elicit secondary ROS generation from biological sources (Leach et al., 2001; Lee et al., 2001). Mitochondria have been indicated as a primary source of ROS during IR (Leach et al., 2001). Specifically, it is suggested that IR-related oxidative stress may be associated with Ca^2+^-dependent cascades and the activation of mitochondrial permeability transition (Leach et al., 2001). However, other researcher suggested that the inhibition of complex I, III or NADPH oxidase (Nox) showed no significant effects on ROS generation, suggesting that mitochondrial electron transport chain or Nox may not contribute to the increase in IR-induced oxidative stress (Rugo et al., 2002). In our study, IR may likely activate mitochondrial permeability transition and compromise antioxidant activity in muscle fibers, subsequently eliciting ROS elevation. IR-induced oxidative stress can be detrimental to muscle cells by altering cellular redox environment and damaging DNA, protein, and lipids (De Lisio et al., 2011; Zuo et al., 2015b). Furthermore, ROS have been implicated in muscle fatigue development due to their critical roles in maintaining calcium hemostasis (Ca^2+^) and force generation (Kandarian and Stevenson, 2002; Moopanar and Allen, 2005). Studies have shown that high levels of ROS may cause Ca^2+^ overload in the sarcoplasm, inducing myocyte necrosis (Wrogemann and Pena, 1976; Powers et al., 2010). We suspect that the IR-induced ROS may play a key role in compromising diaphragm function via the mechanisms of apoptotic activation and Ca^2+^ overload. This is based on our observation that ROS overproduction appeared before the significant decline in muscle function. Apart from oxidative damage, additional mechanisms have been proposed to explain IR-induced muscle weakness (Cairoli et al., 1982; Phelan and Gonyea, 1997; Barton-Davis et al., 1999; Hojan and Milecki, 2014). It is suggested that IR-related muscle injuries are contributed by a combination of direct injuries of myocytes, disturbance of metabolic system, and impairment of microcirculatory environment (Cairoli et al., 1982). For instance, radiation fibrosis syndrome is shown to cause progressive complications that affect muscle function years after RT. Although skeletal muscle is considered radio-resistant relative to other tissues, IR may induce progressive fibrosis in nearby tendons, blood vessels, nerves, which can adversely affect muscle activities (Hojan and Milecki, 2014). Both high and low (within the therapeutic range) dosages of irradiation have been reported to induce acute and delayed muscle necrosis, respectively (Cairoli et al., 1982). Furthermore, irradiation is linked with an immediate loss of electrolytes balance, which has also been implicated in the radiation-associated muscle weakness. For example, IR has been found to alter membrane permeability by inhibiting the active transport of K^+^, thus markedly affecting muscle contraction (Cairoli et al., 1982). High irradiation doses (e.g., 25 Gy) can impede the division of satellite cells and subsequently lower the muscle regenerative capacity (Phelan and Gonyea, 1997; Barton-Davis et al., 1999).

We noted that there is a slight difference between the intact diaphragm and C2C12 cells regarding the pattern of ROS production. On day 3, extracellular ROS formation was markedly elevated within the diaphragm muscle (Figure 3) but was maintained at normal levels in C2C12 cells (Figure 4). This difference may be attributed to the complex physiological structures of skeletal muscles apart from muscle fibers. The non-muscular structures (e.g., capillaries) could also be sensitive to IR and are potentially involved in the complicated ROS generation mechanisms within the intact muscle (Gavin et al., 2005; Rodemann and Blaese, 2007). Furthermore, muscle contraction can be another important factor that contributes to ROS formation. In our experiments, ROS release in diaphragm was measured after 5-min contraction, while the C2C12 cells were not stimulated during the measurement. Contraction–induced ROS may explain why we observed higher ROS levels in the muscular system than in the cellular system on day 3. Moreover, it was suggested by Brander et al. (1997) that radiation may lead to more pronounced diaphragmatic damage *in vivo* than in the *in vitro* models. This is because IR-induced bilateral phrenic nerve impairment is excluded from the isolated cell models, which, may be responsible for the diaphragm weakness observed in patients who received RT (Brander et al., 1997). Therefore, both *in vivo* and *in vitro* studies are valuable when evaluating diaphragm performance in response to radiation exposure in future studies.

Considering the critical roles of ROS in mediating muscle injury, administration of exogenous antioxidants may offer critical protection to diaphragm tissue that is subject to IR exposure (Mutlu-Turkoglu et al., 2000; Robbins and Zhao, 2004; Lu et al., 2006). The potential of antioxidants to attenuate cell damage induced by radiation has been investigated for more than 60 years. Results from animal studies suggest that nutritional antioxidants including vitamin E and selenium (Se) can provide protection against radiation-induced toxic effects (Breccia et al., 1969; Weiss and Landauer, 2003). For instance, Krolak et al. reported a significantly higher survival rate in mice that are treated with normal levels of vitamin E as dietary supplementation than in the control mice on a diet of minimal vitamin E intake after receiving 7.5 Gy IR. A combination usage of various antioxidants can yield optimal outcomes (Weiss and Landauer, 2003). Türkoğlu et al. proposed that vitamin E and Se combination markedly decreased radiation-induced lipid peroxidation. They further suggested that vitamin E and Se administration tends to restore antioxidants to normal levels in radiated tissues (Mutlu-Turkoglu et al., 2000). In addition, many phytochemicals, such as melotonin and genistein have been shown to exert radioprotection on tissues *in vivo* (Weiss and Landauer, 2003). However, there is controversy regarding the use of antioxidant in reducing IR-induced tissue injury because several studies have shown that the antioxidant application during or after RT may compromise the cancer treatment efficiency (Lee et al., 2001; Rugo et al., 2002). In a double-blind trial conducted by Bairati et al., 540 patients with neck or head carcinomas received either a supplementation of tocopherol and β-carotene or placebo during and after IR treatment for 3 years (Lee et al., 2001). The study showed that patients who received antioxidant supplementation experienced less acute negative effects from IR compared to the placebo group. However, a higher local recurrence rate of the neck or head tumor was also observed in the supplemental group (Lee et al., 2001). Our data suggest that it is necessary to develop protective strategies to attenuate IR-induced oxidative stress in diaphragm. Although previous research on antioxidant interventions has shown some positive results, antioxidants should be administrated with caution during RT in terms of dose applied, site of delivery, time and duration of treatment. The development of associated antioxidant strategies that can attenuate the adverse effects of IR on healthy tissues without sacrificing the tumor killing efficacy will be one of the interesting topics for future study. Furthermore, the established physical models to quantify ROS production related to IR will provide useful data for optimizing the doses of antioxidant administration. Future research should be conducted to determine the efficacy of antioxidants supplementation for preventing IR-induced diaphragm dysfunction.

Currently, IR-induced side effects on the diaphragm have been largely overlooked (Hsieh et al., 2016). We found that mice diaphragm muscle exhibits functional damage and cell death in response to 10 Gy IR, which is much lower than the prescription dose of IR treatment for most human cancers (Kong et al., 2006; Moran et al., 2014). Elevated ROS production was observed in both muscle tissue and C2C12 cell cultures following IR, and was suspected to be responsible for the diaphragm dysfunction in IR group. Considering the critical roles of ROS in mediating muscle function, more attention should be paid to alleviate IR-induced diaphragm stress by controlling ROS levels. For example, antioxidant supplementation could be considered for patients receiving high doses of irradiation on the thoracic area. An exploration on the potential correlation between muscle function and ROS levels may facilitate the development of pharmacological treatments for alleviating radiation stress on the diaphragm. Further research is needed to evaluate the long-term radiation effects on the diaphragm in response to a variety of IR doses via physical modeling as well as an examination on the protective effects of antioxidants administration on the diaphragm after RT.

## Author contributions

Conception and design of research: LZ, LL, and SW. Performed experiments: LZ, TZ, and LL. Analyzed data: LZ, TZ, and LL. Interpreted results of experiments: LZ, TZ, and LL. Contributed reagents/materials/analysis tools: LZ, LL, and SW. Prepared figures: LZ, TZ, and LL. Drafted manuscript: LZ, TZ, and LL. Edited and revised manuscript: LZ, TZ, LL, and SW. Approved final version of manuscript: LZ, TZ, LL, and SW.

### Conflict of interest statement

The authors declare that the research was conducted in the absence of any commercial or financial relationships that could be construed as a potential conflict of interest.
